# Generation of a Triadin KnockOut Syndrome Zebrafish Model

**DOI:** 10.3390/ijms22189720

**Published:** 2021-09-08

**Authors:** Vanilla Martina Vecchi, Marco Spreafico, Alessia Brix, Anna Santoni, Simone Sala, Anna Pistocchi, Anna Marozzi, Chiara Di Resta

**Affiliations:** 1 Department of Medical Biotechnology and Translational Medicine, Università degli Studi di Milano, LITA, Segrate, 20090 Milan, Italy; vanilla.vecchi@unimi.it (V.M.V.); marco.spreafico@unimi.it (M.S.); alessia.brix@unimi.it (A.B.); anna.marozzi@unimi.it (A.M.); 2 UOC Clinical Genomics, IRCCS San Raffaele Hospital, 20132 Milan, Italy; santoni.anna@hsr.it (A.S.); diresta.chiara@hsr.it (C.D.R.); 3 Department of Cardiac Electrophysiology and Arrhythmology, IRCCS San Raffaele Hospital, 20132 Milan, Italy; sala.simone@hsr.it

**Keywords:** Triadin KnockOut Syndrome, heart defects, zebrafish, arrhythmic drugs

## Abstract

Different forms of sudden cardiac death have been described, including a recently identified form of genetic arrhythmogenic disorder, named “Triadin KnockOut Syndrome” (TKOS). TKOS is associated with recessive mutations in the *TRDN* gene, encoding for TRIADIN, but the pathogenic mechanism underlying the malignant phenotype has yet to be completely defined. Moreover, patients with TKOS are often refractory to conventional treatment, substantiating the need to identify new therapeutic strategies in order to prevent or treat cardiac events. The zebrafish (*Danio rerio*) heart is highly comparable to the human heart in terms of functions, signal pathways and ion channels, representing a good model to study cardiac disorders. In this work, we generated the first zebrafish model for *trdn* loss-of-function, by means of *trdn* morpholino injections, and characterized its phenotype. Although we did not observe any gross cardiac morphological defect between *trdn* loss-of-function embryos and controls, we found altered cardiac rhythm that was recovered by the administration of arrhythmic drugs. Our model will provide a suitable platform to study the effect of *TRDN* mutations and to perform drug screening to identify new pharmacological strategies for patients carrying TRDN mutations.

## 1. Introduction

Sudden cardiac death (SCD) is a major global public health burden accounting for about 6% of all deaths per year [1]. Defined as “sudden and unexpected death occurring within an hour of the onset of symptoms, or occurring in patients found dead within 24 h of being asymptomatic [2]”, SCD is fatal for up to 5000 young people between 1 and 35 years in the United States of America alone [3]. Up to one third of SCD patients do not present any structural alteration of the heart. Instead, rare inherited arrhythmia syndromes are implicated, such as Long QT Syndrome (LQTS) and Catecholaminergic Polymorphic Ventricular Tachycardia (CPVT). These disorders, manifesting as syncope or arrhythmic episodes, are associated with genetic alterations in ion channels or related proteins, leading to dysregulation in the ionic currents that determine the morphology and duration of the cardiac action potential [4].

Recent studies identified causative mutations in *TRDN* gene (OMIM #603283), encoding TRIADIN protein, in a small number of pediatric cases with overlapping features of LQTS and CPVT [5,6,7,8,9], a condition termed “Triadin KnockOut Syndrome” (TKOS) [10]. TKOS is characterized by a severe and potentially lethal arrhythmogenic phenotype with early onset. Patients with TKOS display clinical features of multiple arrhythmia syndromes, including exercise-induced cardiac arrest in early childhood. In addition, some patients show mild skeletal myopathy or proximal muscle weakness. To date, 23 patients with TKOS have been identified, most of whom carrying mutations determining complete loss of TRIADIN [10,11,12,13].

TRIADIN is a multiprotein family comprising three isoforms that arise from alternative splicing of the *TRDN* gene transcript, namely Trisk 96, Trisk 51 and Trisk 32. Trisk 96 and Trisk 51 are expressed in the skeletal muscle tissue, whereas Trisk 32 is mainly expressed in cardiac muscle cells [14,15,16,17,18]. TRIADIN is a single-pass transmembrane protein of the sarcoplasmic reticulum (SR), consisting of an N-terminal cytosolic domain, a 20-amino-acid-long transmembrane domain embedded in the junctional sarcoplasmic reticulum (jSR) and a C-terminal domain protruding into SR lumen. The three isoforms share the cytoplasmic and transmembrane domains, whereas the SR lumen domain differs in terms of length [14,17,18]. TRIADIN interacts with ryanodine receptor, junctin and calsequestrin, creating the sarcoplasmic reticulum calcium release complex (CRC) and regulating calcium release during excitation–contraction coupling [6,14,19].

*Trdn* knock-out mouse models suffered from cardiac arrhythmias and skeletal muscle weakness, thus indicating that Triadin is involved in cardiac and skeletal muscle function [20,21]. Though the pathogenic mechanism is still not completely understood, recent findings pointed out a Triadin role in maintaining CRC structure, as the reduced expression of CRC proteins was observed in *Trdn*-null mice [20].

Currently, only 21 patients from 16 unrelated families are included in the International Triadin KnockOut Syndrome Registry (ITKOSR) [10]. TKOS is caused by recessively inherited homozygous or compound heterozygous null variants in *TRDN*-encoded cardiac triadin. To date, 17 unique pathogenic variants or likely pathogenic variants have been identified in *TRDN*. The vast majority of these variants are either frameshift, nonsense, or splice site-altering variants that lead to an early stop codon and nonsense-mediated decay. Notably, two pathogenic missense variants (p.T59R and p.T59M), resulting in an unstable protein that undergoes proteasomal degradation, and a large copy number variant deletion of exon 2, have been observed [22].

In particular, our group previously described a severe TKOS form of a 2-year-old boy, who was resuscitated from sudden cardiac arrest [12]. The patient showed a positive family history for SCD by inheriting a novel homozygous variant (c.167T > C, p.Leu56Pro) by the asymptomatic parents, which were heterozygous for the same variant. We hypothesized that the mutation affects protein mobility, leading to the cardiac electrical instability and the severe malignant arrhythmia [12], instead of TRIADIN expression or stability.

Often, conventional pharmacological strategies generally adopted for other forms of arrhythmias (i.e., β-blockers) were ineffective in TKOS patients and the only available treatment is the implantation of a cardioverter defibrillator [10] to prevent the malignant arrhythmogenic episodes. Considering the pediatric age of TKOS patients, the identification of new and less invasive pharmacological strategies is of the utmost importance. Thus, a model for *TRDN* loss-of-function allowing large-scale screening aimed at identifying new candidate drugs to treat TKOS patients would be of great clinical utility.

Zebrafish is a suitable model to study cardiovascular disorders. Indeed, even though its heart is two-chambered, functional and electrical properties, as well as those involved in genes and signaling pathways, are highly conserved between zebrafish and mammals. 

In this work, we assessed the spatio-temporal expression of *trdn* during zebrafish embryo development and we generated a zebrafish model of *trdn* loss-of-function by means of morpholino (MO) injection. We characterized skeletal muscle and heart phenotype in *trdn*-MO-injected embryos, and we observed a dysregulation of cardiac function in terms of a reduction of heart beats. Such a defect in the heart rate was partially recovered by the injection of the wild-type form of the human *TRDN* and treatment with conventional arrhythmic drugs.

We propose this model as a platform to characterize specific *TRDN* mutations and their pathogenic mechanisms and to perform high-throughput drug screening in zebrafish in order to identify candidate drugs for patients with TKOS.

## 2. Results

### 2.1. Zebrafish Trdn Identification and Spatio-Temporal Expression Analyses

To take advantage of zebrafish as a model to investigate *trdn* loss-of-function, we initially characterized its expression during development. Zebrafish *trdn* is present in a single copy on chromosome 20 (nucleotide position: 40,150,612–40,231,379). As in humans, also in zebrafish *trdn* gene encodes for three different splicing products.

**Figure 1:**
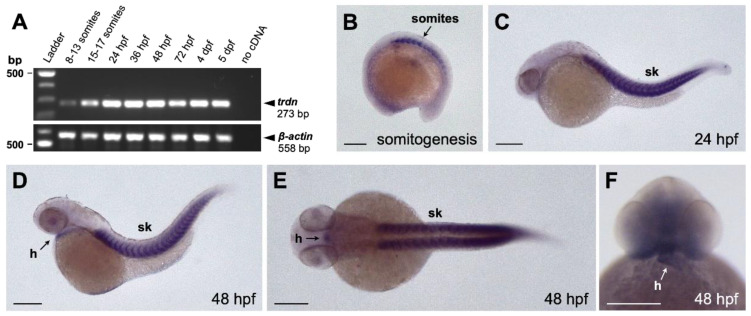
Expression analysis of *trdn* in zebrafish embryo. (**A**) RT-PCR performed on RNA isolated from zebrafish embryos at different developmental stages. *β-actin* was used as the loading control. (**B**–**F**) WISH analyses with a *trdn*-specific probe in zebrafish embryos. *trdn* is expressed in skeletal muscles and heart. (**B**–**D**) Lateral view of a 13 somite (**B**, anterior to the left), 24 hpf (**C**) and 48 hpf (**D**) embryos. (**E**,**F**) Dorsal (**E**) and frontal (**F**) view of a 48 hpf embryo. Scale bars indicate 100 μm. sk, skeletal muscle; h, heart.

We characterized *trdn* expression during embryo development by means of RT-PCR and whole mount in situ hybridization (WISH) analyses. RT-PCR revealed that *trdn* is expressed from somitogenesis up to 5 days post fertilization (dpf, Figure 1A). By WISH analysis, we detected *trdn* mRNA in the somites during somitogenesis (Figure 1B) and in the skeletal muscle from 24 h post-fertilization (hpf, Figure 1C). At 48 hpf, *trdn* was expressed both in the skeletal muscles and in the heart (Figure 1D–F).

These data indicate that, as in humans and mice, zebrafish *triadin* is expressed in both skeletal muscle and heart.

### 2.2. Generation and Characterization of a Trdn Loss-of-Function Model in Zebrafish

**Figure 2:**
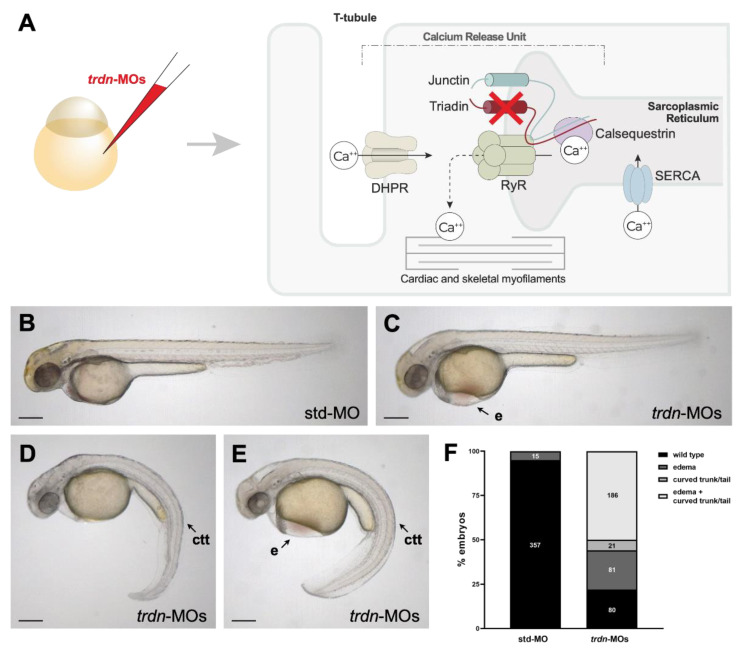
Phenotypical analysis of embryos with *trdn* loss-of-function. (**A**) Schematic representation of Triadin interactions network and *trdn* knockdown strategy. RyR: ryanodine receptor; DHPR: dihydropyridine receptor; SERCA: sarco-endoplasmic reticulum calcium ATPase (**B**–**E**) Representative bright field images of the phenotype of 48 hpf embryos injected with std-MO and *trdn*-MOs. (**F**) Classification of the phenotypes obtained with *trdn*-MOs injection. Values indicate the number of embryos for each phenotype. Scale bars indicate 100 μm. e, edema; ctt, curved trunk/tail.

To ablate the function of *trdn* in zebrafish, we injected zebrafish embryos at 1- to 2-cell stage with two *trdn* morpholinos (MOs), designed to target the translational start site (ATG-MO) and the exon 1—intron 1 junction (splice-MO). To assess the dose-dependent effects, each MO was initially injected at three different concentrations: 0.3, 0.6 and 1.2 pmol/embryo. We evaluated the efficacy of splice-MO and ATG-MO by RT-PCR and Western blot analyses, respectively. Both MOs were efficient and showed a dose-dependent effect (Figure S1A–C). We then assessed the effect of *trdn* knockdown by evaluating the phenotype of zebrafish embryos injected with a cocktail of splice-MO and ATG-MO (*trdn*-MOs), 0.6 pmol/embryo each (Figure 2A). Since *trdn* was expressed in the heart and in the skeletal muscle, we investigated the efficacy of *trdn* knockdown at 48 hpf, a stage in which zebrafish heart is functional and the first skeletal myogenic wave has already been completed [23,24]. Embryos injected with *trdn*-MOs presented cardiac edema and curvature of the trunk/tail region. We used these defects as a benchmark to classify *trdn*-MO-injected embryos in four phenotypical classes (Figure 2B–F): class 1 embryos presenting a morphology similar to the std-MO-injected controls (Figure 2B), class 2 embryos with edema (Figure 2C), class 3 embryos with curved trunk and tail (Figure 2D), and class 4 embryos with both edema and curved trunk and tail (Figure 2E). These alterations were specific to *trdn* knockdown, as they were present in a dose-dependent manner when embryos were injected with increasing concentrations of either ATG-MO or splice-MO alone (Figure S1D,E).

### 2.3. The General Morphology and Organization of Skeletal Muscle Fibres Are Not Impaired by Trdn Knockdown

**Figure 3:**
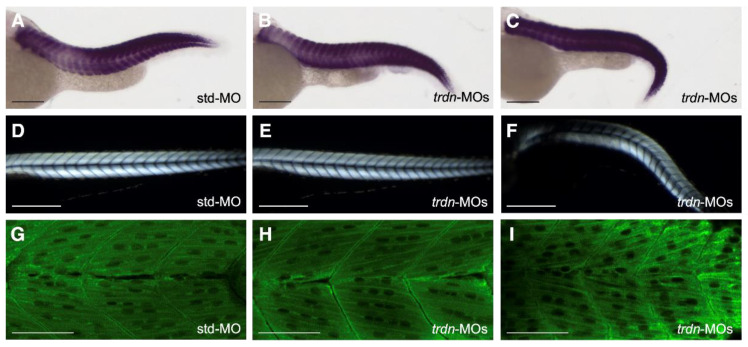
Skeletal muscle morphology evaluation. (**A**–**C**) Representative images of WISH analysis using a *ckma* probe in std-MO (**A**), class 1 wild-type-like (**B**) and class 3 affected (**C**) *trdn*-MO-injected embryos at 48 hpf. (**D**–**F**) Representative birefringence images of std-MO (**D**), class 1 wild-type-like (**E**) and class 3 affected (**F**) *trdn*-MO-injected embryos at 3 dpf. (**G**–**I**) Representative MF20 immunofluorescence images of std-MO (**G**), class 1 wild-type-like (**H**) and class 3 affected (**I**) *trdn*-MO-injected embryos. (**A**–**F**) Scale bars indicate 100 µm. (**G**–**I**) Scale bars indicate 50 μm.

**Figure 4:**
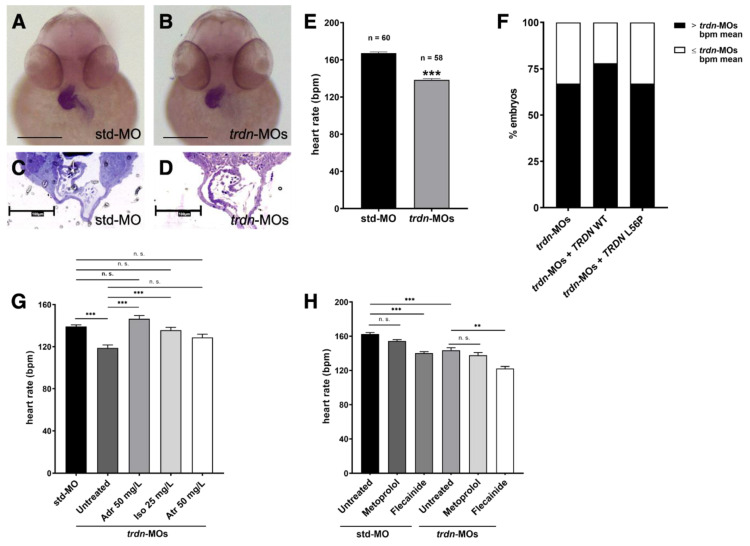
Heart morphology and function evaluation. (**A**,**B**) Frontal view of 48 hpf std-MO and *trdn*-MOs embryos hybridized with *cmlc2* probe using WISH technique. Scale bars indicate 100 μm. (**C**,**D**) Semithin section of std-MO and *trdn*-MOs embryos in the heart region. Scale bars indicate 100 μm. (**E**) Quantification of heartrate in embryos at 48 hpf injected with std-MO and *trdn*-MOs. (**F**) Percentage of embryos with a heartbeat superior to the mean of the heartbeat (bpm) of *trdn*-MOs. (**G**) Heartbeat count in 3 dpf std-MO and *trdn*-MOs untreated or adrenaline-, isoprenaline- and atropine-treated embryos. (**H**) Heartbeat count in 3 dpf std-MO and *trdn*-MOs untreated or treated with metoprolol or flecainide. (**F**–**H**) At least 30 embryos were analyzed for each group. Values are expressed as mean ± SEM. *** *p* < 0.001, ** *p* < 0.01, n.s. = not significant, Mann–Whitney test (**E**) and one-way ANOVA followed by Tukey post-hoc correction (**G**,**H**). Adr, adrenaline; Iso, isoprenaline; Atr, atropine.

*Trdn*-MO-injected embryos presented defects in their trunk/tail region, which might be due to defects in skeletal muscles. To assess the morphology of skeletal muscles, we took advantage of different techniques: by WISH analysis using a probe for *creatine kinase muscle a* (*ckma*)*,* by birefringence assay and by immunofluorescence. WISH experiments revealed no evident differences in skeletal muscles morphology between std-MO-injected embryos and both class 1 (wild-type-like) and class 3 (affected *trdn*-MO-injected embryos) (Figure 3A–C). By birefringence analysis, we did not observe skeletal muscle lesions in *trdn*-MO-injected embryos in comparison to controls (Figure 3D–F). Moreover, immunofluorescence analyses with an anti-sarcomeric myosin antibody (MF20) did not show gross alterations of myosin fibers in *trdn*-MO-injected embryos in comparison to controls (Figure 4G–I).

Taken together, these results indicate that *trdn* knockdown does not affect the overall skeletal muscle tissue structure.

### 2.4. trdn Loss-of-Function Impaired the Heart Rhythm

Since patients with TKOS and *Trdn* KO mice present heart function dysregulation and we observed cardiac edema in our *trdn*-MO-injected embryos, we analyzed both heart morphology and function at the stage of 48 hpf. To analyze heart looping and structure, we performed WISH analysis using a probe for *cardiac myosin light chain 2* (*cmlc2*), a heart-specific marker expressed throughout both the ventricular and atrial portions of the heart. WISH experiments revealed no cardiac structural alterations in *trdn*-MO-injected embryos compared to std-MO-injected embryos (Figure 4A,B). Additionally, semithin sections did not show evident morphological alterations between heart chambers of *trdn*-MO-injected embryos in comparison to controls (Figure 4C,D).

By contrast, *trdn*-MO-injected embryos showed a significant reduction (*p* < 0.0001) in heart rate in comparison to std-MO-injected embryos (Figure 4E, Videos S1 and S2), thus indicating a dysregulation of cardiac electrical function. To assess if the reduced heartbeat presented by the *trdn*-MO-injected embryos might be specifically due to Triadin loss-of-function, we co-injected *trdn*-MOs with wild-type (*trdn*-MOs + *TRDN* WT) or L56P mutant (*trdn*-MOs + *TRDN* L56P) human cardiac isoform of *TRDN* mRNAs. To note, the human *TRDN* gene is only partially conserved with the zebrafish *trdn,* but the mutated amino acid in L56 is present (Figure S2A). To choose the appropriate dose to perform rescue experiments, we injected zebrafish embryos with increasing doses of *TRDN* mRNA alone. We selected the dose of 25 pg/embryo to perform rescue experiments as higher mRNA doses were toxic and induced lethality (Figure S2B). Although not statistically significant, we observed a partial rescue of the heart rate in *trdn*-MOs + *TRDN* WT and, to a lesser extent, in *trdn*-MOs + *TRDN* L56P embryos (Figure S2C). Moreover, when considering the percentage of *trdn*-MOs + *TRDN* WT embryos with a heart rate higher than the mean of the heartbeat of *trdn*-MOs, this was increased to 78%, in comparison to the 67% of *trdn*-MOs. By contrast, *trdn*-MOs + *TRDN* L56P embryos showed no differences (67%) in comparison to *trdn*-MOs embryos (Figure 4F).

Then, to assess if the *trdn*-MOs heart phenotype could be pharmacologically recovered, we treated the embryos with three different arrhythmic drugs (adrenaline, atropine and isoprenaline). At the stage of 3 dpf, embryos were treated with 50 mg/L adrenaline for 30 min, 50 mg/L atropine for 100 min or 25 mg/L isoprenaline for 100 min and then the heartbeat was assessed. The analysis revealed a significant recovery of the heart rate by treatment with adrenaline (Video S3) (untreated *trdn*-MOs vs. *trdn*-MOs + 50 mg/L adrenaline, *p* < 0.0001) and isoprenaline (untreated *trdn*-MOs vs. *trdn*-MOs + 25 mg/L isoprenaline, *p* = 0.0001) in *trdn*-MO-injected embryos. As for atropine, we observed a partial rescue of the heartbeat, even though it did not reach the statistical significance (untreated *trdn*-MOs vs. *trdn*-MOs + 50 mg/L atropine, *p* = 0.0865) (Figure 4G).

Since β-blockers and sodium channel blockers therapy represent the first line treatment of patients with TKOS, we tested metoprolol and flecainide. As expected, in the std-MO-injected embryos, flecainide treatment reduced the heartbeat (untreated std-MO vs. std-MO + flecainide, *p* < 0.0001). Interestingly, also in *trdn*-MO-injected embryos, we obtained a reduction (untreated *trdn*-MO vs. *trdn*-MO + flecainide, *p* = 0.0011) of cardiac frequency due to the pharmacological effects of the anti-arrhythmic drug, suggesting its efficacy also in the absence of Triadin function (Figure 4H, Video S4). As for metoprolol, it did not affect the heartbeat in a significant manner even though we observed a slight reduction both in std-MO (untreated std-MO vs. std-MO + metoprolol, *p* = 0.9999) and in *trdn*-MO-injected embryos (untreated *trdn*-MO vs. *trdn*-MO + metoprolol, *p* = 0.9999) (Figure 4H).

## 3. Discussion

TRIADIN is a family comprising three single-pass transmembrane proteins arising from alternative splicing of a single *TRDN* gene, expressed specifically in skeletal and cardiac muscle tissues [14,15,16]. TRIADIN was demonstrated to be involved in the maintenance of the CRC, playing a role in excitation-contraction coupling modulation [6,14,19]. However, its precise function is still to be deeply characterized. Recent studies identified *TRDN* mutations in patients affected by a disorder presenting overlapping features of CPVT and LQTS, termed as TKOS [10]. Precise pathomechanisms underlying TKOS are still largely unknown and their treatment with conventional antiarrhythmic drugs showed no efficacy; hence, implantation of a cardioverter defibrillator is the only suitable approach [10,13]. Indeed, pharmacological treatment would be more advisable, especially considering that TKOS occurs at a pediatric age.

Zebrafish is widely adopted as a model to study heart physiology and related disorders [23]; therefore, it would represent a useful tool to study the effects of *trdn* dysregulation. Moreover, it represents a suitable system to perform drug screening in order to identify a possible pharmacological approach for patients with TKOS. However, there is still a lack of knowledge about *trdn* function and expression in zebrafish.

Here, we generated and characterized a zebrafish model for *trdn* loss-of-function. First, we assessed *trdn* spatio-temporal expression in zebrafish embryos, confirming the previous findings (available online: https://zfin.org/ZDB-PUB-040907-1 (accessed on 25 July 2021). As in human, mouse and other animal models [25,26], we observed *trdn* expression specifically in the skeletal muscle and in the heart, at developmental stages ranging from somitogenesis to 5 dpf.

We then performed *trdn* knockdown in zebrafish embryos by means of co-injection of a cocktail of *trdn* MOs. All identified patients with TKOS carry mutations in the shared portion of protein among the three isoforms [10,13], often resulting in complete loss of cardiac and skeletal muscle TRIADIN. Therefore, we used both an ATG-MO and a splice-MO targeting exon 1-intron 1 splice site to achieve the knockdown of all the three zebrafish *trdn* isoforms. *trdn*-MO-injected embryos presented an altered morphology with edema and curvature of the trunk/tail region. Importantly, we did not observe any difference in terms of lethality between std- and *trdn*-MO-injected embryos, similarly to *Trdn^−/−^* mice, which showed no embryonic lethality [21,27]. The morphant embryo phenotype was variable, as edema and curvature of the trunk/tail region were observed both individually and concurrently. It is worth noting that such variability was observed also in patients with TKOS, of whom only six were reported to present skeletal myopathy in addition to cardiac events to date [10]. Indeed, the percentage of *trdn*-MOs embryos presenting curvature of the trunk/tail region (~56%) was high, in comparison to patients with TKOS (<30%). This discrepancy can be due to multiple reasons. First, the number of available patient’s clinical records is still limited compared to the high number of analysed zebrafish embryos; therefore, a comprehensive characterization of TKOS phenotype in human is yet to be defined. Additionally, the high variability of the TKOS phenotype might veil more subtle skeletal muscle impairments. Moreover, there is no information regarding *TRDN* mutations in patients affected by myopathy without heart function dysregulation. In this regard, we observed few *trdn*-MO-injected embryos showing defects in trunk/tail without presenting cardiac edema, thereby raising the possibility that *TRDN* loss might rarely affect skeletal muscle without involvement of the heart muscle. Indeed, more studies are needed to better understand the molecular basis of TKOS phenotype variability and future whole-genome sequencing analyses in myopathy patients will possibly help to clarify this point.

To assess the feasibility of our *trdn* knockdown zebrafish as an in vivo TKOS model, we characterized both the skeletal muscle and heart phenotype of *trdn*-MOs embryos. By multiple techniques, we revealed that the overall structure of skeletal muscle is not grossly compromised in *trdn*-MO-injected embryos. Similarly, also heart formation, looping and chamber organization were not altered. These data are consistent with previous studies in a *Trdn^−/−^* mouse model that presents no gross morphological heart and skeletal muscle alterations [20,21,27]. Despite the general morphology of the heart being unaffected, *trdn*-MO-injected embryos showed a dysregulation of cardiac function with reduced heart rate in comparison to control embryos. Although *TRDN* loss is associated with tachycardia in patients with TKOS, in *Trdn^−/−^* mice [20], the electrocardiogram (ECG) evaluation demonstrated sinus bradycardia and Triadin overexpression was proven to predispose rat cardiac myocytes to arrythmia [28]. Thus, *TRDN* loss-of-function might determine a reduced heart contraction capability, which eventually predisposes to tachycardia disorders through still undefined mechanisms. Supporting this hypothesis, two patients with TKOS were reported to suffer from in utero bradycardia [5,11]. To assess the specificity of the *trdn*-loss-of function on the heart rate, we injected the wild-type form of the human cardiac isoform of *TRDN*. The wild-type *TRDN* mRNA determined a partial, but not statistically significant, rescue of the heartbeat presented by *trdn*-loss-of-function embryos. To note, the amino acid conservation of the human and zebrafish TRIADIN was only around 30% and the human mRNA might compensate only in part the loss of the zebrafish endogenous protein. Alternatively, the dose of human *TRDN* mRNA was not sufficient to recover the phenotype. Unfortunately, the injection of higher doses of WT *TRDN* mRNA was toxic for the embryos. The injection of the mutated cardiac isoform carrying the mutation previously identified in a patient with TKOS [12] was not efficient in recovering the bradycardia of *trdn*-loss-of-function embryos. Indeed, this mutation has been reported to affect protein mobility but not its expression and stability [12], possibly resulting in a gain-of-function phenotype instead of a loss-of-function. Further analyses will be necessary to better elucidate this point.

Despite reduced heart rate, the *trdn*-MOs embryo heart was still functional and sensitive to stimuli, as treatment with different arrythmia drugs was able to recover the heart rate to a level comparable to control embryos. Interestingly, *trdn*-MOs embryos were also sensitive to metoprolol, a β-blocker that is used for TKOS patients treatment [10].

In conclusion, our *trdn*-deficient zebrafish model will represent a useful tool to investigate *trdn* physiological role and the effects of its loss. Indeed, in comparison to mouse, zebrafish allows for an easy-to-perform in vivo evaluation of heart function. For example, transparency of zebrafish embryos allows a rapid evaluation of heartbeat, which can represent an easy and cheap read-out to perform high-throughput drug screening. Additionally, finer analyses, such as the evaluation of myocardial wall velocity, blood flow, regularity of heartbeat and fractional area changes (reviewed by [23]), could be applied to obtain more insight into the precise effects of *TRDN* on heart function. In addition, *trdn* knockdown could be performed in zebrafish transgenic lines, such as *Tg(vmhc:eGFP)* [29] and *Tg(amhc:eGFP)* [30] labelling ventriculum and atrium, respectively, to discriminate precise triadin functions in different heart regions. Importantly, by microinjection of specific mRNAs, our model will also represent a useful platform to validate the causative *TRDN* mutations identified in patients with TKOS.

## 4. Materials and Methods

### 4.1. Animals

Zebrafish embryos were collected by natural spawning. Embryos were staged as described in Kimmel et al. and raised in fish water (Instant Ocean, 0.1% Methylene Blue) at 28 °C in Petri dishes, according to established techniques [31]. Embryonic ages are expressed in hours post-fertilization (hpf) and days post-fertilization (dpf). To prevent pigmentation, 0.003% 1-phenyl-2-thiourea (PTU, Sigma-Aldrich, St. Louis, MO, USA) was added to the fish water. Embryos were anesthetized with 0.016% tricaine (Ethyl 3-aminobenzoate methanesulfonate salt, Sigma-Aldrich) before proceeding with experimental protocols.

### 4.2. Microinjection

For loss-of-function experiments, two antisense morpholinos (MOs, Gene Tools, Philomath, OR, USA) targeting *trdn* translational start site (ATG-MO) and exon 1—intron 1 junction (splice-MO) were designed to knockdown the zebrafish *trdn* transcript. MO sequences were *trdn* ATG-MO 5′-TCCATCTCTCTCATGCACTAACAGG-3′ and *trdn* splice-MO 5′-ATGAAGTTCACAGTACCTTCCATCT-3′. MOs were injected into 1- to 2-cell stage embryos at the concentrations of 0.3, 0.6 and 1.2 pmol/embryo in 1× Danieau buffer (pH 7.6). When co-injected, they were used at the concentration of 0.6 pmol/embryo each. Identical amounts of a standard MO (std-MO, Gene Tools), targeting human *HBB* (5′-CCTCTTACCTCAGTTACAATTTATA-3′), was used as a control for all injections.

For rescue experiments, human wild type or L56P mutant TRISK 32 full-length mRNA were injected (25 pg/embryo). mRNAs were in vitro transcribed by using the mMESSAGE mMACHINE T7 kit (Thermo Fisher Scientific, Waltham, MA, United States) from pcDNA3.1 +-TRISK32 WT or pcDNA3.1 +-TRISK32 L56P plasmids (Eurofins Genomics Srl, Vimodrone, Milan, Italy).

### 4.3. RT-PCR

Total RNA was isolated from at least 30 zebrafish embryos using NucleoZOL reagent (Macherey-Nagel, Düren, Germany) according to the manufacturer’s protocol. A measure of 1 μg of RNA was treated with RQ1 RNase-free DNase (Promega, Madison, WI, USA) and then reverse transcription (RT) reaction was performed with GoScript Reverse Transcription Kit (Promega), as specified by the manufacturer’s instructions. PCR was performed by using the GoTaq DNA polymerase (Promega) and the following primers: *trdn* fw 5′-CTGGACCCGGGGAATTGACT-3′; *trdn* rev: 5′-CTGGACCCGGGGAATTGACT-3′; β-actin fw 5′-TGTTTTCCCCTCCATTGTTGG-3′; *β-actin* rev 5′-TTCTCCTTGATGTCACGGAC-3′. *trdn* primers were designed to amplify a region shared by all the *trdn* isoforms. A total of 25 μL PCR reactions containing 0.8 μM of each primer, 0.1 mM dNTPs, 0.125 μL of GoTaq and 2 μL of cDNA were prepared. PCR protocol was the following: 95 °C for 3 min; 35 cycles of 95 °C for 10 s, 58 °C (*trdn*) or 60 °C (*β-actin*) for 30 s and 72 °C for 10 s; 72 °C for 5 min. PCR products were loaded and resolved on 1% agarose gel. SYBR Safe DNA Gel Stain (Thermo Fisher Scientific) was used to visualize DNA bands. A 100 bp DNA Ladder (Promega) was used as a size marker.

### 4.4. Western Blot

Total proteins were extracted in RIPA buffer (10 mM Tris-HCl pH 8.0, 1 mM EDTA, 0.5 mM EGTA, 1% Triton X-100, 0.1% sodium deoxycholate, 0.1% SDS, 140 mM NaCl) with the addition of protease inhibitor cocktail (Roche, Basel, Switzerland) from at least 50 zebrafish embryos at 48 hpf, 2 μL/embryo. Lysates were sonicated at 30 Hz 3 times for 5 s. Then, they were centrifuged 10 min at 16,000× *g* at 4 °C. The supernatant was recovered, and extracts were quantified by using the Quantum Micro Protein Assay (EuroClone, Pero, Italy). A total of 40 μg of proteins were loaded in a 10% acrylamide/polyacrylamide gel and subjected to electrophoresis. Proteins were transferred onto polyvinylidene fluoride (PVDF) membranes that were incubated in blocking solution (5% skimmed powder milk in TBS containing 0.1% Tween-20) for 1 h at room temperature (RT) before o/n incubation at 4 °C with primary antibodies diluted in blocking solution. Membranes were then incubated for 1 h at RT with HRP-conjugated secondary antibody diluted in blocking solution. Protein bands were detected by using a WESTAR ECL detection system (Cyanagen, Bologna, Italy). Images were acquired with the Alliance MINI HD9 AUTO Western Blot Imaging System (UVItec Limited, Cambridge, UK) and analysed with the related software. Tubulin was used as the internal control. Primary antibodies were mouse anti-TRDN (1:500, T3569, Sigma-Aldrich) and mouse anti-tubulin (1:2500, T9026, Merck-Millipore, Burlington, MA, USA). Secondary antibody was HRP-conjugated horse anti-mouse (Cell Signaling Technology, Danvers, MA, USA) 1:4000 and 1:8000 for tubulin and triadin detection, respectively. Anti-TRDN antibody was specific for the longest TRDN isoform and was chosen as it was previously shown to possess reactivity against zebrafish Trdn [32].

### 4.5. Imaging

Digital brightfield and birefringence images of living embryos were captured using a microscope equipped with a digital camera with LAS Leica Imaging software version 4.13 (Leica, Wetzlar, Germany). Birefringence was performed by setting anesthetized embryos on a polarizing filter and placing a second polarizing filter over the objective lens as previously described [33,34]. Embryos were oriented to maximize the brightness of the trunk and tail through the crossed filters. Images were processed using Adobe Photoshop software version 22.2.0.

### 4.6. Whole-Mount In Situ Hybridization (WISH)

To synthetize the *trdn* probe, *trdn* cDNA was amplified by PCR reaction using the following primers: *trdn* fw 5′-CTGGACCCGGGGAATTGACT-3′; *trdn*-T7 rev: 5′-GCGTAATACGACTCACTATAGGGCATCCTCCTCTTCTTCCGGC-3′. *trdn* primers were designed to amplify a region shared by all the *trdn* isoforms. PCR protocol was the same as described in the RT-PCR section. The probe was synthetized by using T7 RNA polymerases kit (Promega) and digoxigenin (DIG) RNA labeling mix (Roche). *cmlc2* (*myl7*, Zebrafish Information Network) and *ckma* probes were previously described [35,36]. To perform WISH analysis, embryos were fixed for 2 h in 4% paraformaldehyde in phosphate-buffered saline (PBS), then rinsed 3 times with PBS, dehydrated in 100% methanol and stored at −20 °C until being processed for WISH [37]. WISH was carried out on at least 15 embryos as previously described [38]. 1 ng/μL DIG-labeled probe was incubated o/n at 65 °C in hybridization mix (50% formamide, 5X Saline Sodium Citrate pH 5, 0.1% Tween-20, 4.6 μL/mL citric acid 1M, 50 μg/mL heparin and 500 μg/mL t-RNA). Digital images of all embryos were captured using a microscope equipped with digital camera with LAS Leica Imaging software (Leica). Images were processed using Adobe Photoshop software.

### 4.7. Immunofluorescence

Zebrafish whole-mount immunostaining was performed as described [39]. The primary antibody was mouse anti-MF20 1:4 (Developmental Studies Hybridoma Bank, Iowa City, IA, USA). The secondary antibody was Alexa Fluor 488-conjugated goat anti-mouse IgG 1:400 (Cell Signaling Technologies).

Images were acquired using an A1R confocal microscope (Nikon, Minato, Tokyo, Japan) with 40× water immersion objective and 488 nm laser. Single stack images of at least five embryos were acquired for each sample. To generate z-stack images, the Z-project tool of the Fiji software was used [40]. For each embryo, 7–9 single stacks were merged using the average intensity projection type.

### 4.8. Semithin Sections

Std-MO and *trdn*-MO zebrafish embryos at 48 hpf were fixed overnight with 2.5% glutaraldehyde/4% paraformaldehyde/cacodylate buffer (pH 7.2) at RT, postfixed in 1% osmium tetroxide, dehydrated in an ethanol series and embedded with epoxy resin. Samples are sectioned with an ultramicrotome. Semithin sections (1 μm) were stained with toluidine blue. Images were acquired using the Hamamatsu slide scanner (OSI optoelectronics, Hawthorne, CA, USA).

### 4.9. Heart Rhythm Detection

The heartbeat frequency was measured in zebrafish embryos by optical visualization, recording videos for 15 s. Heartbeats were manually counted, and the number of beats/15 s was multiplied by 4 to obtain the beats per minute (bpm) [41]. To minimize the ambient temperature effect on the zebrafish embryo heart rate, 5 embryos per time were taken from the 28 °C incubator to be assessed for their heartbeat.

### 4.10. Drug Treatment

Zebrafish embryos at 48 hpf or 3 dpf were manually dechorionated and placed in 24-well plates, 15 embryos per well.

For arrhythmic drug treatment, embryos were treated with 50 mg/L adrenaline (Galenica Senese S.r.l., Monteroni d’Arbia, Siena, Italy) for 30 min, 25 mg/L isoprenaline (S.A.L.F. S.p.A., Cenate sotto, Bergamo, Italy) for 100 min or 50 mg/L atropine (S.A.L.F. S.p.A.) for 100 min in a total volume of 1 mL of fish water. Treatments were performed at 3 dpf, as it is a stage in which zebrafish embryos are responsive to such drugs [42]. The concentration for each drug was chosen as the most efficient among three different doses in increasing heartbeat in control embryos (Figure S3).

For anti-arrhythmic drug treatment, 100 mg metoprolol (Recordati S.p.A., Milan, Italy) and flecainide (DOC Generici S.r.l., Milan, Italy) tablets were dissolved in water, 16.5 mg/mL and 13 mg/mL, respectively. A measure of 10 µL of dissolved metoprolol or flecainide was added in a total volume of 1 mL. Embryos at 48 hpf were treated o/n and the heartbeat was assessed at 3 dpf.

### 4.11. Statistical Analysis

Each experiment was performed at least three times. Statistical analyses were performed with GraphPad Prism software version 8.0.2 (La Jolla, CA, USA; Available online: www.graphpad.com (available on 30 August 2021)). The Gaussian data distribution of all datasets was assessed by Shapiro–Wilk or Kolmogorov–Smirnov normality test. Mann–Whitney test was used when comparing two groups. When comparing more than two groups, one-way ANOVA followed by Tukey post-hoc correction was used for normally distributed datasets whereas Kruskal–Wallis followed by Dunn’s multiple comparisons test was used for non-normally distributed datasets, as indicated in figure legends. For Kruskal–Wallis test, mean and interquartile range (IQR) values are indicated in the related figure legends.

## 5. Conclusions

In conclusion, by generation of a *trdn*-deficient zebrafish model, in this work, we demonstrated that *trdn* loss in zebrafish determines a phenotype similar to *Trdn*-null mice and patients with TKOS. Our model will provide a useful tool for future studies aiming at better understand *TRDN* function and will serve as a platform for the identification of novel candidate drugs for TKOS treatment.

## Data Availability

Data will be available upon request.

## Supplementary Materials

The following are available online at https://www.mdpi.com/article/10.3390/ijms22189720/s1.
