# Supplementary material for: Generation of a Triadin KnockOut Syndrome Zebrafish Model
Source: Int J Mol Sci. 2021 Sep 8;22(18):9720. doi: 10.3390/ijms22189720 (PMC8471218; doi:10.3390/ijms22189720)
Supplement: Supplementary file 1 [file ijms-22-09720-s001.zip › ijms-1353961-SI.pdf]

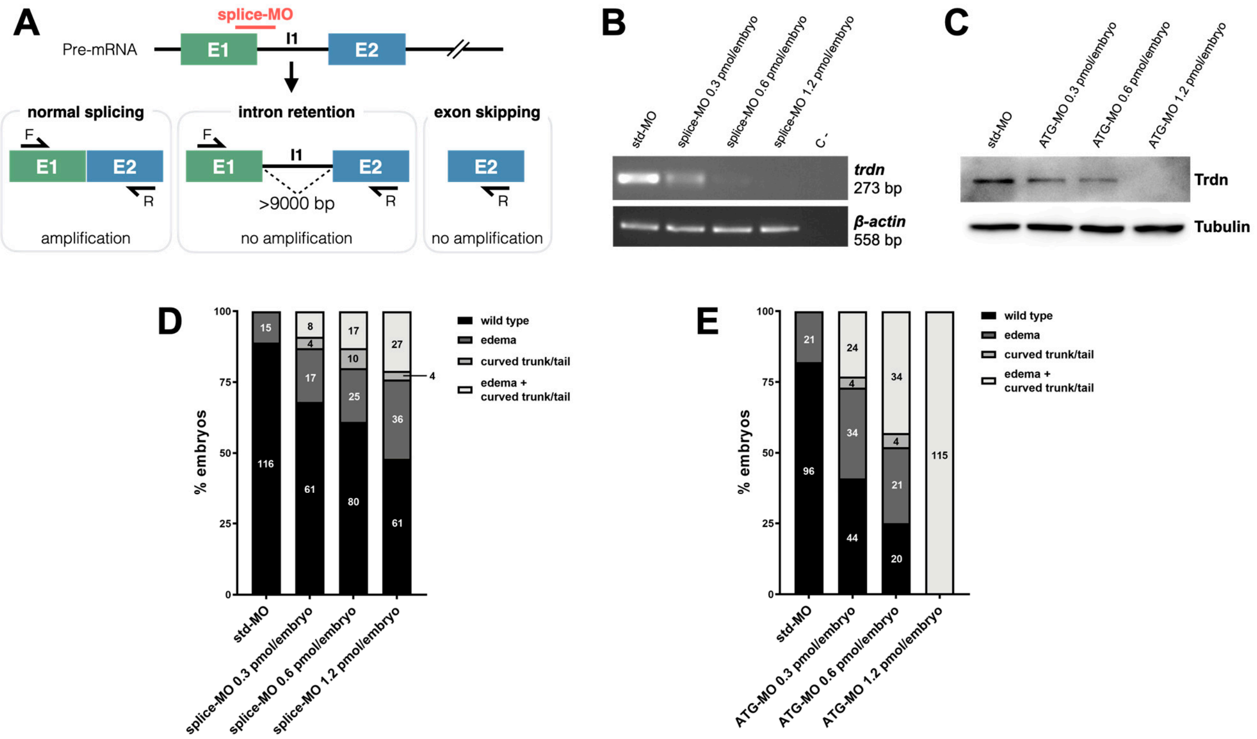

Figure S1. Validation of *trdn*-MOs efficacy and dose-dependency assays. (**A**) Schematic representation of splice-MO validation by RT-PCR. To assess dose-dependent effect of splice-MO, RT-PCR was performed by using forward and reverse primers annealing to *trdn* exon 1 and exon 2, respectively, so that both exon 1 skipping and intron 1 (> 9000 bp length) retention determined no amplification. F and R arrows show the position of forward and reverse primers, respectively. E1, exon 1; E2, exon 2; I1, intron 1. (**B**) RT-PCR performed on RNA extracted from zebrafish embryos injected with std-MO or *trdn* splice-MO at different concentrations (0.3, 0.6, 1.2 pmol/embryo). Zebrafish *β-actin* was used as an internal control in each sample. (**C**) Western blot analysis of Trdn expression in zebrafish embryos injected with std-MO or *trdn* ATG-MO at different concentrations (0.3, 0.6, 1.2 pmol/embryo). Tubulin was used as internal control in each sample. (**D-E**) Quantification of embryos phenotypes following *trdn* splice-MO (D) and ATG-MO (**E**) injection. Values indicate the number of embryos for each phenotype.

A

```
TRDN_HomoSapiens        MTEITAEGNASTTTTVIDSKNGSVPKSPGKVLKRTVTEDIVTTFSSPAAWLLVIALIITW   60
Trdn_DanioRerio         -----MEARSTTTTTIVEAKN-DVSSHSVRSSKRTMTDDLYSTFSSPMAWILVLALIITW   54
                              *..::****::::** .* .   :  ***:*:*: :***** **:**:******

TRDN_HomoSapiens        SAVAIVMFDLVDYKNFSASSIAKI---------------GSDPLKLVRDAMEETTDWIYG   105
Trdn_DanioRerio         SAVAIIMFDLLDSKGLEGPQPHRVRKTLKEAGSLRGGIHHISPEKAVSEAMDDSSDWITS   114
                        *****:****:* *.:.. .  ::                 .* * * :**::::*** .

TRDN_HomoSapiens        FFSLLSDIISSEDEEDDDGDEDT-DKGEIDEPPLRKKEIHKDKTEKQEKPERKIQTKVTH   164
Trdn_DanioRerio         ILTFMTNLVAPEEEEDEGERHFVKKKGEFLPPRKKVTEIWAKEMLEEE--GEYLE-----   167
                        :::::::::: *:***:.  . . .***:  *  : .**  .:  ::*   . ::

TRDN_HomoSapiens        KEKEKGKEKVREKEKPE----KKATHKEKIEKKEKPETKTLAKEQKKAKTAEKSEEKTKK   220
Trdn_DanioRerio         DEEEEEEEEMDTDEEEEDYEEEEEEYDEELEEEEEE-EEELEEEEEEE-LDEEEEEELEK   225
                        .*:*: :*::  .*: *    ::  :.*::*::*:   : * :*:::    *:.**: :*

TRDN_HomoSapiens        EVKGGKQEKVKQTAAKVKEVQKTPSKPKEKEDKEKAAVSKHEQKGQSPAIPPPLPTEQAS   280
Trdn_DanioRerio         E-----------------------------------------------------------   226
                        *

TRDN_HomoSapiens        RPTPASPALEGKYFFFS   297
Trdn_DanioRerio         -----------------   226
```

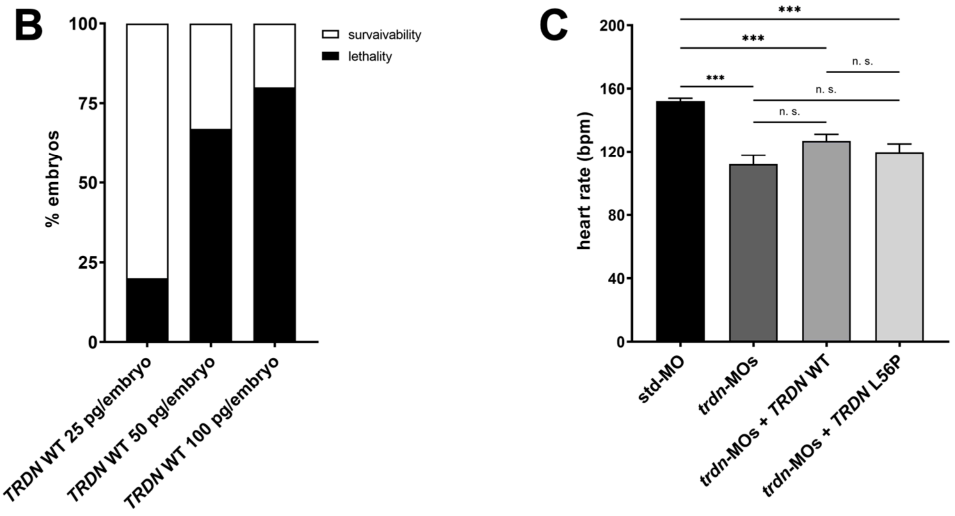

Figure S2. Human *TRDN* injection in zebrafish embryos. **(A)** Human and zebrafish cardiac Triadin amino acid sequences alignment. Asterisks represent fully conserved residues; colons indicate positions with conservation between amino acid groups of similar properties; periods indicate positions with conservation between amino acid groups of weakly similar properties. Mutated amino acid in the mutant human Triadin (L56P) is indicated in red. (**B**) Quantification of embryos lethality following human *TRDN* WT mRNA injection at different doses. (**C**) Quantification of heartrate in embryos at 48 hpf injected with std-MO, *trdn*-MOs, *trdn*-MOs + *TRDN* WT and *trdn*-MOs + *TRDN* L56P. At least 30 embryos were analyzed for each group. Values are expressed as mean ± SEM. Std-MO: mean = 152, IQR = 12; *trdn*-MOs: mean = 110, IQR = 44; *trdn*-MOs + *TRDN* WT: mean = 127, IQR = 20; *trdn*-MOs + *TRDN* L56P: mean = 120, IQR = 44. ***p < 0.001, n.s. = not significant, Kruskal-Wallis followed by Dunn's multiple comparisons test.

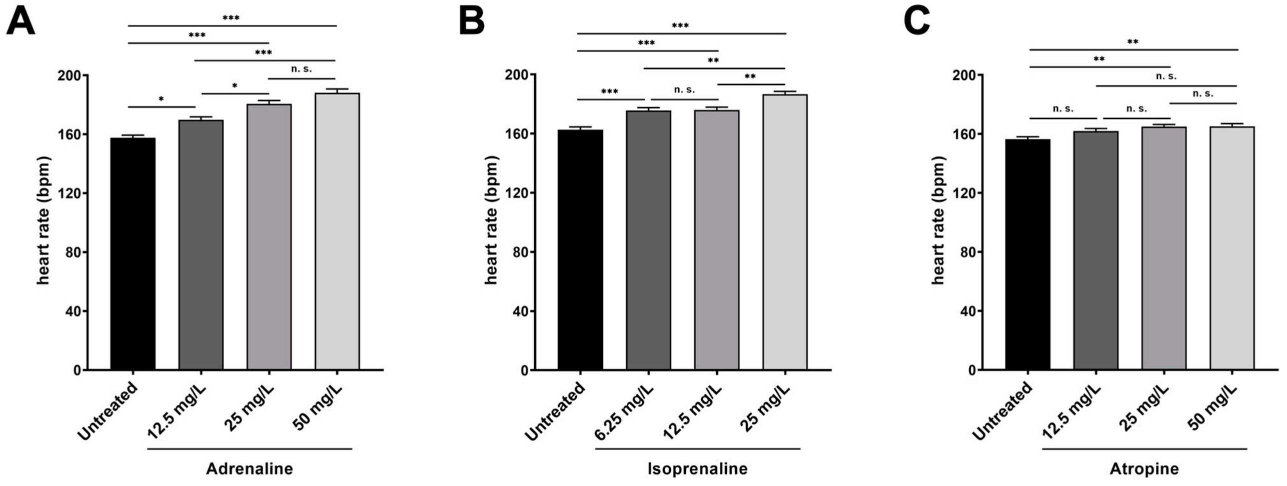

Figure S3. Dose-effect test for adrenaline, isoprenaline and atropine. Heartbeat count in 3 dpf zebrafish embryos untreated or treated with increasing doses of adrenaline (**A**), isoprenaline (**B**) and atropine (**C**). At least 30 embryos were analyzed for each group. Values are expressed as mean ± SEM. (**A**) Untreated: mean = 158, IQR = 12; 12.5 mg/L adrenaline: mean = 170, IQR = 20; 25 mg/L adrenaline: mean = 181, IQR = 20; 50 mg/L adrenaline: mean = 188, IQR = 13. *p < 0.05, ***p < 0.001, n.s. = not significant, Kruskal-Wallis followed by Dunn's multiple comparisons test. (**B**) Untreated: mean = 163, IQR = 8; 6.25 mg/L isoprenaline: mean = 176, IQR = 13; 12.5 mg/L isoprenaline: mean = 176, IQR = 13; 25 mg/L isoprenaline: mean = 187, IQR = 16. **p < 0.01, ***p < 0.001, n.s. = not significant, Kruskal-Wallis followed by Dunn's multiple comparisons test. (**C**) **p < 0.01, n.s. = not significant, one-way ANOVA followed by Tukey post-hoc correction.
